# First Wave of the 2016-17 Cholera Outbreak in Hodeidah City, Yemen - ACF Experience and Lessons Learned

**DOI:** 10.1371/currents.outbreaks.5c338264469fa046ef013e48a71fb1c5

**Published:** 2017-10-13

**Authors:** Mathias Altmann, Miguel Suarez-Bustamante, Celine Soulier, Celine Lesavre, Caroline Antoine

**Affiliations:** Expertise and Advocacy Department, Action Contre la Faim, Paris, France; Expertise and Advocacy Department, Action Contre la Faim, Paris, France; Expertise and Advocacy Department, Action Contre la Faim, Paris, France; Expertise and Advocacy Department, Action Contre la Faim, Paris, France; Expertise and Advocacy Department, Action Contre la Faim, Paris, France

## Abstract

**Introduction:**

Although cases were reported only in 2010 and 2011, cholera is probably endemic in Yemen. In the context of a civil war, a cholera outbreak was declared in different parts of the country October 6th, 2016. This paper describes the ACF outbreak response in Hodeidah city from October 28th, 2016 to February 28th, 2017 in order to add knowledge to this large outbreak.

**Methods:**

The ACF outbreak response in Hodeidah city included a case management component and prevention measures in the community. In partnership with the Ministry of Public Health and Population of Yemen (MoPHP), the case management component included a Cholera Treatment Center (CTC) implemented in the Al Thoraw hospital, 11 Oral Rehydration Therapy Corners (ORTCs) and an active case finding system. In partnership with other stakeholders, prevention measures in the community, including access to safe water and hygiene promotion, were implemented in the most affected communities of the city.

**Results:**

From October 28th, 2016 until February 28th, 2017, ACF provided care to 8,270 Acute Watery Diarrhea (AWD) cases, of which 5,210 (63%) were suspected cholera cases, in the CTC and the 11 ORTCs implemented in Hodeidah city. The attack rate was higher among people living in Al Hali district, with a peak in November 2016. At the CTC, 8% of children under 5 years-old also presented with Severe Acute Malnutrition (SAM). The Case-Fatality Rate (CFR) was low (0.07%) but 15% of admitted cases defaulted for cultural and security reasons. Environmental management lacked the information to appropriately target affected areas. Financial resources did not allow complete coverage of the city.

**Conclusion:**

Response to the first wave of a large cholera outbreak in Hodeidah city was successful in maintaining a CFR <1% in the CTC. However, considering the actual context of Yemen and its water infrastructure, much more efforts are needed to control the current outbreak resurgence.

## Introduction

Cholera remains a major public health risk in the WHO Eastern Mediterranean Region. During the last decade, at least 14 out of 22 countries in the region have reported cholera cases, often in epidemic proportions. Countries in this region facing complex emergencies are particularly at risk, as they lack safe drinking-water and sanitation facilities and management. The full extent of the burden of cholera in the region is difficult to estimate due to weak surveillance systems in some endemic countries, in addition to underreporting of cases. Nevertheless it is estimated that the number of suspected cases may be around 188,000 per annum.^1^ Explosive outbreaks of cholera have been reported from Afghanistan, Djibouti, Iraq, Pakistan, Sudan, Somalia and Yemen in the last decade.^2^ In Yemen, cholera outbreaks were reported as early as the 1980s,^3^ in 2010 and 2011, with 300^4^ and 31,789 cases,^5^ respectively. Spatial modelling technique defined Yemen as an endemic country for Cholera with around 17,546 cases per year and a Case Fatality Rate (CFR) of 3.20%.^6^

Since 2011, Yemen is facing a complex situation, with about 5 years of a political conflict and over 18 months of a civil war worsened by an economical blockage. Over a population of circa 28 million people,^7^ there are more than 2 million internally displaced people (IDPs),^8^ 462,000 children with Severe Acute Malnutrition (SAM), 14.5 million people (half of its population) without access to safe drinking water and 14.8 million with no access to health care services (only 45% of health facilities are functional).^9^ In this context, a cholera outbreak is a significant threat for the country.

On October 6th, 2016, Yemen’s Ministry of Public Health and Population (MoPHP) officially declared the epidemic, with 11 cases in Sana’a and four cases in Al-Bayda governorate showing positive laboratory tests with Vibrio cholera 01, serotype Ogawa.^10^ Some days after, the first suspected cholera cases in the Governorate of Hodeidah, a Northern, coastal, region of the country were reported.^11^

To respond to the outbreak, the Governorate Health Officer (GHO) and the WaSH Sub-Cluster requested support to operational health and water, sanitation and hygiene (WaSH) partners in Hodeidah. The international non-governmental organization Action Contre la Faim (ACF) took the lead with the sub-national health cluster in coordinating the response with other NGOs in Hodeidah city. This paper describes ACF’s experience in the outbreak response, presents some descriptive results and highlights operational constraints.

## Methods

***Preparation of the outbreak response***

The geographical area of the intervention was Hodeidah city, capital of the Al Hudaydah Governorate. Hodeidah is the fourth-largest city in Yemen with a population of around 400,000 people,^12^ a low-density spread over approximately 86 square kilometers^13^ and separated into three districts: Al HaliHali, Al Hawak and Al Mina (Figure 1). By mid-October 2016, ACF staff had carried out a rapid assessment of the situation to determine how ACF could contribute to a consistent and effective outbreak response. This activity was conducted by interviewing key informants, reviewing official statistics, visiting health facilities and affected communities, as well as by meeting with local health authorities to identify their needs. Following the assessment, a comprehensive outbreak response was developed by ACF staff in Hodeidah city.

***Outbreak response***

ACF outbreak response in Hodeidah city included a case management component and prevention measures (WaSH) in the communities (Figure 1). In partnership with the MoPHP, ACF provided support to Hodeidah Governorate in running cholera and/or Acute Watery Diarrhea (AWD) case management structures. The MoPHP provided physical space and key staff. ACF provided support in the construction and/or rehabilitation of health centers, staff recruitment (nurses, cleaners, pharmacist, logisticians, WaSH workers), supervision and training, chain supplying of drugs and medical material, set up of support systems (logistics, WaSH, data entry and analysis), Human Resource management, financial resources to roll-out the intervention and clinical supervision.

**ACF and other NGOs areas of intervention, Hodeidah city, Yemen, October 2016- March 2017.**
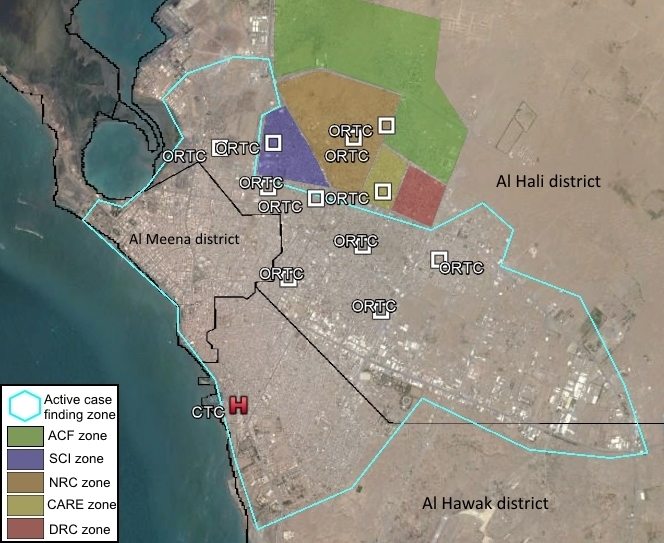
ACF: Action Contre la Faim; SCI: Save the Children International; NRC: Norwegian Refugee Council; DRC: Danish Refugee Council

Case management included interventions at three levels:

- Secondary health care level intervention: Cholera Treatment Center (CTC)

- Primary health care level intervention: Oral rehydration therapy corners (ORTCs)

- Community level intervention: active case finding

***Cholera Treatment Center (CTC)***

In Al Thowra hospital, the biggest hospital of the city, ACF set up a CTC with a 60 bed capacity (see H in Figure 1). This facility provided 24/7 services for 4 months (from October 28th, 2016 to February 28th, 2017) free of charge for the patients. CTC activities included triage, emergency rehydration and close monitoring until recovery. Case management of patients followed WHO protocols.^14^

*Admission criteria*

The criterion for admission was as follows: all patients with 3 or more watery stools per day (a medium to large volume, not necessarily “rice water”). Fever and/or vomiting were not used as criteria. Patients with other types of diarrhea were not admitted.

*Epidemiological definition*

The suspected cholera case definition was “a patient aged 5 years or more who develops AWD, with or without vomiting, in an area where there is a cholera epidemic”.^15^ This WHO definition was used considering all cases presented in an outbreak context that started on October 6th, 2016 when the first cholera cases were confirmed by lab tests.

*Classification*

Clinical signs of dehydration (sunken or dry eyes, tears, dry mouth/tongue, thirst, skin pinch, radial pulse and blood pressure) were used, as recommended by WHO guideline,^16^ to classify dehydration level by mild, moderate and severe. Shock was defined as blood pressure inferior to 60/40, a weak or absent pulse and signs of hypodynamic circulation. SAM cases were children under 5 years of age diagnosed with Mid-Upper Arm Circumference (MUAC) < 115 mm.

*Treatment*

Depending on the dehydration severity, three treatment plans were applied:

- Plan A for no/mild dehydration: ORS packets to take home after 2 hours observation

- Plan B for moderate dehydration: ORS packets at the center

- Plan C for severe dehydration: Intravenous (IV) fluid Ringer's lactate solution.

A nutrition corner, with ORS, ReSoMal and milk, was set up for SAM children. SAM children under-five with mild dehydration were considered as common viral diarrhea cases and were treated with ReSoMal at a slow rhythm, according to regular protocols for rehydration of SAM children.^17^ Moderate and severe dehydration cases in the same group were considered as cholera cases and were treated by ORS, according to WHO recommendations.^18^

***Oral Rehydration Treatment Corners (ORTCs)***

ORTCs are spaces (sometimes rooms, sometimes corners) located in primary health care facilities that are able to provide Oral Rehydration Solution (ORS) to AWD/cholera patients with mild to moderate dehydration and to refer severe cases to the CTC. ACF response included 11 ORTCs (one in a hospital and 10 in health centers) in Al Hali district (Figure 1), which was the most affected by the outbreak. In these spaces, patients received ORS until recovery following WHO rehydration treatment plan. Severe cases were transferred to the CTC. Activities started from November 13th, 2016.

***Active Case Finding***

Active case finding was implemented in the areas not covered by any WaSH interventions (Al Hawak and Al Mena districts; Figure 1). Four epi teams, including three people each, were trained and deployed to identify the level of dehydration in AWD cases. Patients’ addresses were collected on a daily basis at the CTC. Streets, blocks, or neighborhoods where the majority of AWD/cholera cases originated from were targeted for active case finding activities. Field teams visited them systematically to identify and provide care to new AWD/cholera cases. When a simple AWD/cholera case was identified, it was given ORS. Complicated cases were referred to the CTC. Field teams also disinfected patients’ households with chlorine and provided special health and WaSH education to families focusing on the proper hygienic practices at household level to prevent disease transmission. Education activities primarily targeted patients’ households; however, patients’ neighbors also received health and WaSH education sessions. Before any visit, and in order to ensure a good population acceptance, local authorities were contacted to obtain permission and support for the work in the neighborhood.

***Preventive measures***

Together with partners (Save the Children International, Norwegian Refugee Council, and Danish Refugee Council), ACF decided to concentrate its interventions on access to safe water and hygiene promotion in the most affected district of the city (Figure 1).

*Access to safe water*

From October 29th, 2016 to the end of February, 2017, ACF implemented water trucking in Al Hali district (green area in Figure 1), to ensure access to a minimum of 7.5 liters/person/day of safe water for drinking and cooking purposes (as per SPHERE standard) to 2,430 households (17,010 individuals). The water was chlorinated directly in the truck, and monitored on a daily basis by ACF team, against the standard of 0.5 to 1 mg/liter Free Residual Chlorine (FRC). Only fiber glass tracks were used as they are better suited to transport water than metallic trucks. Furthermore, ACF also rented big vans on which plastic tanks were installed. Twelve water communal distribution points were also rehabilitated/ constructed to increase the number of taps available, and reduce queuing time at collection points. Water quality was routinely monitored at water source (0.5 to 1 mg/liter) and at household level (0.2 mg/liter) (among water tanks users) to assess the risk of post-collection contamination.

81 Community Health Worker were identified and intensively trained to further provide hygiene education in the targeted communities. They also were considered to lead and support the community during the community solid waste management campaign. For this activity, small incentives were given for their transportation and communication cost.

*Hygiene promotion*

Water supply activities were supported by hygiene promotion, focusing on appropriate hand-washing and waste disposal practices. ACF recruited a team of 12 hygiene promoters who conducted group sensitization (2,097 people) and home visits (25,704 people) to disseminate essential messages to prevent the spread of AWD. This team also provided training and regular supervision to a network of 79 Community Health Volunteers, to increase outreach of hygiene promotion activities, and ensure continuity beyond ACF intervention. Mass sensitization was organized at water points during water trucking (22,439 people).

***Coordination mechanism***

From the onset of the cholera outbreak, ACF coordinated its response with the sub-national Health and WASH Clusters in Hodeidah. The WASH response was also closely coordinated with the local provider of water supply and sanitation, which manages the water network serving the targeted population of Al Hali. ACF produced weekly cholera situation and activity reports providing updates on both the WASH and Health responses, which were shared with sub-national and national Health and WASH Clusters. ACF also bi-weekly shared updates on its cholera response at the Inter Cluster Coordination Meeting.

***Ethics Statement***

This assessment was part of an operational public health intervention and thus did not undergo institutional review board review. The data used in this study were retrospectively collected and analyzed and were fully anonymized before the authors had access to them. Only secondary information that was collected for monitoring surveillance purposes was used for the analysis. No specific informed consent was obtained.

## Results

ACF provided care to 8,270 AWD cases, of which 5,210 (63%) were suspected cholera cases treated in the CTC and the 11 ORTCs in Hodeidah city. This corresponds to an overall attack rate (AR) of 130 per 10,000 people over the study period (October 28th, 2016 to February 28th, 2017).

***CTC***

*Patient’s characteristics*

From October 28th, 2016 until February 28th, 2017, 4,517 AWD cases were admitted at the CTC. Of these, 3,070 (68%) were suspected cholera cases. Number of admission was at the highest at the CTC opening and decreased from January onwards (Figure 2).

**Figure:**
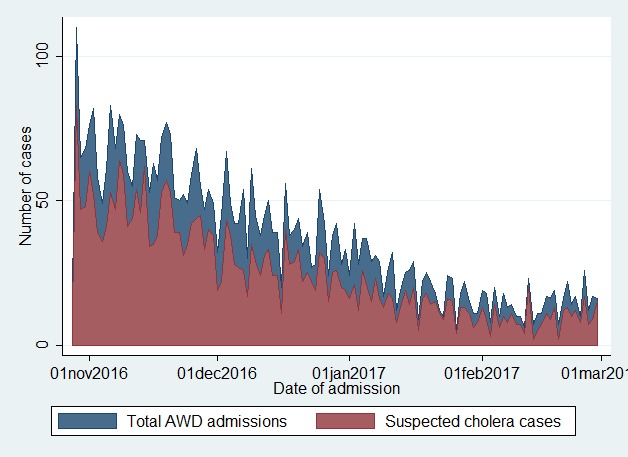
**Fig. 2:** Number of AWD cases, by date of admission at the Al Thowra hospital, from October 28, 2016 to February 28, 2017.

The origin of the AWD cases was mainly Hodeidah city (58% from Al Hali district, 30% from Al Hawak and 7% from Al Mina). Less than 5% of the patients came from districts and villages surrounding the city. The characteristics of the cases are displayed in Table 1. Median age was 11 years. 27.3% of the admissions were children between 1 and 5 years of age. Among children less than 5 years old, eight percent had also severe acute malnutrition and 17.8% were severely dehydrated. Among SAM children, 33.9% were under 1 year old; 39.1% and 56.5% were moderately and severely dehydrated cases, respectively. Overall, 25% of admitted cases had episodes of vomiting during their stay.

**Table:** **Table 1:** Characteristics of 4516 AWD cases admitted to the CTC in Al Thowra hospital, 28th of Oct. 2016 – 28th of Feb. 2017

Characteristics	n/N	%
Age (years)		
< 1	211/4517	4.7
[1-5[	1236/4517	27.4
[5-14[	1140/4517	25.2
>=14	1930/4517	42.7
Sex, male	2245/4517	49.7
Severe Acute Malnutrition	115/1447	8.0
Diarrhea		
watery	4481/4517	99.2
Rice watery	36/4517	0.8
Dehydration level		
mild	1595/4517	35.3
moderate	2104/4517	46.6
severe	818/4517	18.1
Shock	115/2528	4.6
Lethargic	75/2528	3.0

*Treatment*

Plan A, B and C was applied respectively to 33.4%, 47.6% and 19.0% of the AWD cases. 65.7% received antibiotics, in addition to Plan B and C. Average length of stay in the CTC was 3.4 hours (SD: 3.5) and 11.4 hours (SD: 11.0) for Plan B and C, respectively.

*Outcomes*

Four deaths occurred at the CTC. Of these, three were older than 5 years old. This corresponds to a CFR at the CTC of 0.07%. 3,768 (83.4%) cases were cured, 53 (1.2%) were transferred to other wards of the hospital after diarrhea stopped, and 691 (15.3%) defaulted. In three of the four fatal outcomes, the death occurred in the first four hours from the admission at the CTC.

## ORTCs

Between November 13th, 2016 and February 28th, 2017, ORTCs received 3,753 admissions, of which 57% were among children under 5. Median age was 4 years (inter quartile range: 2-11). Both sexes were equally affected. Over 90% of the cases were mild cases and 9% were moderate. At discharge, 97% of the cases were cured, 1.8% defaulted and about 1% of the cases were transferred to the CTC due to severe dehydration.

***Active Case Finding***

As February 28th, 2017, the teams had visited over 7,598 households since mid-December 2016. On average, 120 to 140 households were visited per day. The teams identified over 700 AWD cases, of which 91 were referred to the CTC.

***Preventive measures***

From practical aspects, as metallic tankers are not adequate to transfer chlorinated water, we tried to exclude the trucks selection to those made of fiber glass. We succeeded but fiber glass tankers were limited and we also rented big van on which we installed 2-3 plastic tanks. We monitored the FRC to ensure trucks remained disinfected. ACF did monitor the chlorine residual in water sources. This strategy proved to be effective in pushing local actors (local corporation for water supply and sanitation) to do this activity, ensuring a more sustainable intervention.

## Discussion

*Timeliness of the intervention*

Our intervention started around two weeks after the first cholera cases were confirmed. However, this was already after the peak of the epicurve. This delay highlights the need to strengthen preparedness, including early detection and prompt response.

*Case definition*

Thanks to the Hodeidah central laboratory, eight cholera cases were officially confirmed in Al Hali, Al Hawak and Al Mina, during calendar week 42 in 2016.^19^ According to the WHO Standard case definition of cholera,^20^ all “AWD cases aged 5 years or more” should have been counted as suspected cases from this date. However, there is no clear definition of what constitutes “an area where there is a cholera epidemic”. Is this a country, a governorate, a district or sub-district? Furthermore, deficiencies in the surveillance system, lack of rapid diagnostic tests, and low coverage of health facilities have most likely contributed to underreporting. It is important to note that the epidemiological definition is more specific, putting emphasis on adult cases and ruling out viral diarrhea cases in children under 5. The clinical definition (i.e. AWD) is more sensitive, as it tries to identify cholera cases based on a syndromic approach and includes children under five, who are particularly at risk of cholera.^21^

*Attack Rate*

Our overall AR was 130 cases per 10,000 people (5,210/400,000). This AR is probably underestimated as cases might have been admitted to other health facilities of MoPHP. Nonetheless, it was much higher than the AR calculated at the country level in the same period, confirming Hodeidah was a hotspot during this first wave. For an estimated countrywide population of 28 million, a cumulative total of 22,456 suspected cholera cases were reported as of March 2017,^22^ corresponding to an overall attack rate of 8 per 10,000 people.

*Case management*

Similar to the overall CFR in the country reported in the same period,^22^ the CFR in our CTC was low (<1%). This might be due to the relative low proportion of patients presenting with severe dehydration at admission. It can also indicate that case management was well conducted. Despite most cases occurred in Al Hali district, Al Thowra hospital proved to be a good choice for the CTC, as most people knew the hospital and care was free of charge. Long distance was not an obstacle in access to the CTC. ORTCs strategy was also effective to reduce mild cases at the CTC.

We had around 15% of defaulters. It was reported that some patients were unsatisfied because they were prescribed ORS and not IV fluids. ORS does not seem to be perceived as a drug. More information and referral to ORTCs should have been suggested for these cases to avoid CTC surcharge. Mothers of sick children, as well as sick women often had to go back home at night due to cultural reasons, insecurity or fears that airstrikes would target the hospital. Patients therefore requested to be discharged. Ensuring safety of humanitarian space could have a direct effect on health outcomes.

Overall, length of stay was low. This was partially due to the low proportion of severe cases. Besides, the short length of stay had the goal of reducing the proportion of defaulters, completing the treatment before patients requested being discharged.

Appropriate cholera kits (IV fluids, ORS and antibiotics) were available thanks to WHO donations. Other drugs for managing complications had to be bought locally, as international purchases would have required several months (>6). Local purchase followed ACF Standard Operating Procedure for the Management of Medical Supplies. Antibiotic were used, both for moderately and severely dehydrated patients. Although WHO cholera treatment protocol recommends antibiotics use only for severe cases,^20^ some authors recommend its use also for moderate cases.^23^ To our knowledge, no information was available on the resistance pattern of the outbreak strain, which would have certified the efficacy of erythromycin and amoxicillin.

At the moment of this cholera outbreak, Yemen was facing a critical food insecurity situation,^9^ with prevalence of both global acute malnutrition (GAM) and of SAM higher than WHO emergency threshold (GAM >15%). In this context, it was not surprising that many of the children admitted to the CTC presented with both dehydration and SAM (around 8%). In comparison, prevalence of SAM in the general population was reported to be 3.3%.^24^ This suggests that both conditions are worsening each other’s. Acute malnutrition was diagnosed using MUAC rather than Weight for Height Z-score (WHZ), because MUAC is less affected by dehydration than WHZ.^25^ However, because both criteria are affected, WHZ was measured to confirm the nutritional status of SAM children at their discharge. Rehydration of severely dehydrated SAM children is difficult, since the capacity of these children to absorb liquid is hampered and they can quickly get over-hydrated. At the same time, they are at risk of dying due to shocks, if they do not receive enough fluids in a short time. ReSoMal is not recommended for children with SAM either with suspected cholera, or with ‘profuse’ watery diarrhea, because of the need to keep up with stool sodium losses. Instead, WHO recommend standard ORS for children with SAM and profuse losses due to cholera.^18^ The implementation of this recommendation is in practice hindered by the uncertainty around both the definition of a cholera case (especially among children younger than 5) and of the term ‘profuse’. Clearer guidance would be helpful to ensure that the different indications for ORS versus ReSoMal are understood by clinicians on the ground and more easily applied.

*Preventive measures*

While cholera outbreaks tend to be associated with the rainy season,^26^ it was not the case in this first wave of the outbreak, which started during the dry season. As no investigation was done, the origin of the outbreak remains unclear.

ACF concentrated its interventions on access to safe water and hygiene promotion in the most affected districts of the city. Lessons learnt from previous cholera responses indicate that the most effective preventive measures are those improving access to safe water, waste disposal, along with mass hygiene promotion campaigns to support hand-washing with soap.^27^ Latrine construction is recognized to be time and resource consuming, and has less immediate impact in the first three months of the response. WASH interventions targeted only Al Hali district as it was the most affected area and financial constraints did not allow for a complete coverage of the city. Furthermore, information on the origin of the cases was poorly shared and coordinated between ORTCs and CTC. This would have improved the targeting of the response.

Oral vaccine was not considered in our response strategy as it was not available in the country and the CFR was low. However, WHO recommends its use in endemic areas, such as Yemen.^28^ Considering the political crisis and the war, vaccination will likely remain not available in the coming months.

*Coordination*

The coordination was done at the city level. In an urban context like Hodeidah city, coordination of activities should be done on smaller geographic areas, such as ORTC catchment areas or blocks. This would facilitate the exchange of information, in particular, on: (i) the most affected neighborhoods; (ii) the areas where people have no access to health facilities; (iii) real time geographical analysis on new admissions in ORTCs and CTC. This would improve rapid detection of new cases and reduce further transmission.

## Current Status

The number of new cholera/AWD cases decreased in Hodeidah city and in the whole country until end of April 2017, and ACF decided to stop its intervention in the CTC. On April 27th, WHO declared the resurgence of the outbreak in many parts of the country.^29^ The outbreak spread to around 210 districts in 18 governorates, and the case fatality rate exceeded 1% in many governorates. This second wave showed that a cholera outbreak can quickly restart and points to the importance of response preparedness in endemic crisis affected contexts.

## Competing Interests

The authors are employed by ACF, who implemented the intervention. The authors have declared that no competing interests exist.

## Data Availability

All relevant data are available from the Dryad repository: doi:10.5061/dryad.738c1 (http://doi.org/10.5061/dryad.738c1).

## Corresponding Author

Dr. Mathias Altmann: maltmann@actioncontrelafaim.org; altmannmathias@yahoo.fr
